# Supplementary material for: Performance of o1 pro and GPT-4 in Self-Assessment Questions for Nephrology Board Renewal
Source: Front Med (Lausanne). 2025 Nov 25;12:1702668. doi: 10.3389/fmed.2025.1702668 (PMC12685630; doi:10.3389/fmed.2025.1702668)
Supplement: Supplementary file 1 [file Supplementary_file_1.pdf]

Supplemental Table S1: Number of questions in each of the four categories included in each year

Category	Exam year 2014	2015	2016	2017	2018	2019	2020	2021	2022	2023	Overall
**Taxonomy**											
Recall	8	8	9	8	9	9	12	10	19	16	108
Interpretations	10	12	7	9	7	4	1	3	0	3	56
Problem-Solving	7	5	4	3	4	7	6	7	1	1	45
**Question Type**											
General Questions	8	8	10	8	9	12	12	10	18	16	111
Clinical Questions	17	17	10	12	11	8	7	10	2	4	98
**Image Inclusion**											
Non-Image Questions	19	17	15	14	14	15	18	17	20	20	169
Image Questions	6	8	5	6	6	5	1	3	0	0	40
**Subspecialty**											
CKD/ESKD	9	7	2	2	4	3	4	5	7	2	45
AKI	0	1	4	2	1	0	1	1	0	0	10
Glomerular Diseases	6	4	5	8	8	6	5	6	3	6	57
Tubulointerstitial Diseases	0	4	1	0	3	2	2	2	2	3	19
Hypertension/Vascular Diseases	3	1	2	1	1	1	2	3	1	3	18
Water/Electrolytes/Acid-Base Disorder	4	3	2	3	1	3	3	2	1	1	23
ADPKD/Urology	3	3	1	0	0	1	1	0	1	2	12
Basic Medicine	0	2	3	4	2	4	1	1	5	3	25

CKD, chronic kidney disease; ESKD, end-stage kidney disease; AKI, acute kidney injury; ADPKD, autosomal dominant polycystic kidney disease.

Supplemental Table S2: The proportion of correct answers of o1 pro and GPT-4 in the Water/Electrolytes/Acid-Base Disorder subspecialty, stratified by taxonomy and question type.

	The proportion of correct answers in the Water/Electrolytes/Acid-Base Disorder		
**Category**	**o1 pro**	**GPT-4**	**p-value**
**Taxonomy**			
Recall	4/5 (80.0%)	3/5 (60%)	1.000
Interpretation	5/9 (55.6%)	6/9 (66.7%)	1.000
Problem-Solving	8/9 (88.9%)	8/9 (88.9%)	1.000
**Question Type**			
General Questions	5/6 (83.3%)	5/6 (83.3%)	1.000
Clinical Questions	12/17 (70.6%)	12/17 (70.6%)	1.000

The performance of o1 pro and GPT-4 is reported for each category of Self-Assessment Questions for Nephrology Board Renewal. Differences in performance between large language models were queried using chi-squared and Fisher's exact tests.

Supplemental Table S3: Online Publication Timeline for Self-Assessment Questions and Answers for Nephrology Board Renewal

Exam Year	Question Publication Date	Answer Publication Date	URL
2014	Publication Date Not Found	Publication Date Not Found	Link
2015	Publication Date Not Found	Publication Date Not Found	Link
2016	Publication Date Not Found	Publication Date Not Found	Link
2017	Publication Date Not Found	Publication Date Not Found	Link
2018	Publication Date Not Found	Publication Date Not Found	Link
2019	Publication Date Not Found	Publication Date Not Found	Link
2020	November 6, 2020	Publication Date Not Found	Link
2021	November 8, 2021	January 11, 2022	Link
2022	November 7, 2022	January 19, 2023	Link
2023	November 10, 2023	January 25, 2024	Link

Question Publication Date: The date on which the self-assessment questions for the corresponding exam year were made available online. Answer Publication Date: Refers to the date when the answers to the self-assessment questions were published online. The phrase "Publication Date Not Found" indicates that the release date could not be determined despite attempts to locate this information. URL: Provides a direct link to the online resource where the questions and answers can be accessed. Each URL was hyperlinked to facilitate easy access.
